# Identifying Hot Spots of Tuberculosis in Nigeria Using an Early Warning Outbreak Recognition System: Retrospective Analysis of Implications for Active Case Finding Interventions

**DOI:** 10.2196/40311

**Published:** 2023-02-08

**Authors:** Chidubem Ogbudebe, Dohyo Jeong, Bethrand Odume, Ogoamaka Chukwuogo, Cyril Dim, Sani Useni, Okey Okuzu, Chenchita Malolan, Dohyeong Kim, Fiemu Nwariaku, Nkiru Nwokoye, Stephanie Gande, Debby Nongo, Rupert Eneogu, Temitayo Odusote, Salewa Oyelaran, Obioma Chijioke-Akaniro, Nrip Nihalani, Chukwuma Anyaike, Mustapha Gidado

**Affiliations:** 1 Technical Division KNCV Tuberculosis Foundation Abuja Nigeria; 2 University of Texas Dallas, TX United States; 3 College of Medicine University of Nigeria Teaching Hospital Enugu Nigeria; 4 InStrat Global Health Solutions Abuja Nigeria; 5 University of Texas Southwestern Medical Center Dallas, TX United States; 6 Office of Human Immunodeficiency Virus/Acquired Immunodeficiency Syndrome and Tuberculosis United States Agency for International Development Abuja Nigeria; 7 National Tuberculosis, Leprosy and Buruli Ulcer Control Program Federal Ministry of Health Abuja Nigeria; 8 Plus91 Technologies Delhi India; 9 Program Management Unit KNCV Tuberculosis Foundation Hague Netherlands

**Keywords:** early warning outbreak recognition system, active case finding, WHO-four-symptom screen, GeneXpert, active case, cluster, early warning, hot spot, mapping, disease spread, infection spread, retrospective study, retrospective analysis, surveillance, outbreak, TB, tuberculosis, infectious disease, case finding, communicable disease

## Abstract

**Background:**

Undiagnosed tuberculosis (TB) cases are the major challenge to TB control in Nigeria. An early warning outbreak recognition system (EWORS) is a system that is primarily used to detect infectious disease outbreaks; this system can be used as a case-based geospatial tool for the real-time identification of hot spot areas with clusters of TB patients. TB screening targeted at such hot spots should yield more TB cases than screening targeted at non–hot spots.

**Objective:**

We aimed to demonstrate the effectiveness of an EWORS for TB hot spot mapping as a tool for detecting areas with increased TB case yields in high TB-burden states of Nigeria.

**Methods:**

KNCV Tuberculosis Foundation Nigeria deployed an EWORS to 14 high-burden states in Nigeria. The system used an advanced surveillance mechanism to identify TB patients’ residences in clusters, enabling it to predict areas with elevated disease spread (ie, hot spots) at the ward level. TB screening outreach using the World Health Organization 4-symptom screening method was conducted in 121 hot spot wards and 213 non–hot spot wards selected from the same communities. Presumptive cases identified were evaluated for TB using the GeneXpert instrument or chest X-ray. Confirmed TB cases from both areas were linked to treatment. Data from the hot spot and non–hot spot wards were analyzed retrospectively for this study.

**Results:**

During the 16-month intervention, a total of 1,962,042 persons (n=734,384, 37.4% male, n=1,227,658, 62.6% female) and 2,025,286 persons (n=701,103, 34.6% male, n=1,324,183, 65.4% female) participated in the community TB screening outreaches in the hot spot and non–hot spot areas, respectively. Presumptive cases among all patients screened were 268,264 (N=3,987,328, 6.7%) and confirmed TB cases were 22,618 (N=222,270, 10.1%). The number needed to screen to diagnose a TB case in the hot spot and non–hot spot areas was 146 and 193 per 10,000 people, respectively.

**Conclusions:**

Active TB case finding in EWORS-mapped hot spot areas yielded higher TB cases than the non–hot spot areas in the 14 high-burden states of Nigeria. With the application of EWORS, the precision of diagnosing TB among presumptive cases increased from 0.077 to 0.103, and the number of presumptive cases needed to diagnose a TB case decreased from 14.047 to 10.255 per 10,000 people.

## Introduction

Tuberculosis (TB) continues to be a global health concern, which explains why ending the disease epidemic by 2030 is a target of sustainable development goals [1]. Regarding the burden of TB at the country level, Nigeria is sixth globally and the first in Africa [2]; therefore, controlling this chronic infectious disease is a top priority for the national TB program [3]. Globally, the number of annual case notifications is usually far below the estimated incident cases. Unfortunately, this gap (ie, missing TB cases), which had consistently narrowed over the years, widened in 2020 due to a large drop in the number of newly diagnosed TB cases due to the impact of the COVID-19 pandemic [2,4]. In 2020, there were about 9.9 million estimated incident TB cases worldwide, but only 5.8 million were notified, leaving a large gap of about 4.1 million missing cases; notably, Nigeria contributed significantly to this global gap, ranking first in Africa [2].

These missing TB cases could either be due to underreporting or underdiagnosis. In Nigeria, however, underdiagnosis contributes to most missing cases; this calls on the country to strengthen access to high-quality screening, diagnostic, and treatment services [5]. The KNCV Tuberculosis Foundation Nigeria (KNCV Nigeria) continues to assist the Nigerian TB control program in this regard through several projects, including the successful demonstration of the excellent capacity of TB loop-mediated isothermal amplification technology for TB diagnosis among adults in Nigeria [6], as well as the need for X-ray–based mass TB screening in Nigerian prisons [7].

Because undiagnosed TB cases are the major challenge to TB control in Nigeria, any strategy that will identify ongoing TB transmission hot spots in the community is crucial for finding and treating missing TB cases. Electronic recording and reporting of TB are becoming adopted widely by the national TB control programs [8]. The electronic data can be imported into a geographic information system (GIS) for mapping and spatial analysis; thus, the data can be used to identify TB transmission hot spots in the community [8]. Such an electronic GIS strategy has been used in early warning systems (EWSs) for infectious disease surveillance [9], such as the China Infectious Disease Automated Alert and Response System (CIDARS) [10] and an early warning outbreak recognition system (EWORS) implemented by the Indonesian Ministry of Health [11]. Both systems serve as complementary country-wide disease surveillance tools; however, while the CIDARS is a case-based EWS, the EWORS in Indonesia is a syndromic surveillance-based EWS [9]. In Nigeria, an EWORS is operational at the subnational level for routine electronic disease surveillance—the system automatically sends elevated disease activity messages to appropriate disease surveillance officers, which triggers the investigation of the affected areas to prevent outbreaks [12].

Though the EWORS is an early-warning system for infectious diseases to detect outbreaks and guide control practice, it can also predict TB hot spots at the community level by identifying TB patients’ residences in clusters. Because TB is infectious, it is expected that community-based active case finding (ACF) in such EWORS-identified hot spot areas will yield more TB cases when compared to non–hot spot areas. To demonstrate this expectation, KNCV Nigeria applied an EWORS as a geospatial tool for real-time identification of hot spot areas with high TB prevalence for targeted community-based ACF interventions. This innovative project aimed to demonstrate the effectiveness of EWORS TB hot spot mapping for increased TB case yield in 14 states of Nigeria with a high TB burden.

## Methods

### Overview

This is a retrospective review of data from community-based active TB screening interventions by KNCV Nigeria in EWORS-identified TB hot spot areas in 14 states of Nigeria with a high TB burden. This public health intervention was part of the US Agency for International Development–funded TB Local Organization Network Regions 1 and 2 Project. The 14 states involved include 8 northern Nigerian states (Bauchi, Benue, Kaduna, Katsina, Kano, Nasarawa, Plateau, and Taraba) and 6 southern Nigerian states (Anambra, Akwa Ibom, Cross River, Delta, Imo, and Rivers). The 16-month intervention lasted from October 2020 to January 2022.

KNCV Nigeria instituted TB hot spot analysis using InStrat Global Health Solutions’ EWORS to inform community mass TB screening interventions in the identified communities in the 14 project states. The EWORS used an advanced surveillance mechanism to identify TB patients’ residences in clusters, enabling it to predict hot spots at the ward and community level. The EWORS hot spot analytic dashboard portal was developed per state. State hot spot data visualization included heat maps that allowed users to drill down to the ward or community level to pinpoint locations of active cases. Every day, EWORS pulled real-time data from a Commcare-based mobile app—an open-source data capture tool running on Android smartphones that records routine TB surveillance data at health facilities providing TB services in the project states. The Commcare cloud server sent data to the EWORS via a custom data transfer application programming interface. Information was synchronized that predicted the likelihood of the occurrence of disease clusters at the ward or community level and included patient-level clinical data (ie, TB symptoms, TB investigations, TB diagnoses, and treatment status) and nonclinical data (ie, state of residence, local government area [LGA], and ward).

The EWORS triggered an alarm when a hot spot was identified. For the project, a hot spot was identified when any ward or community had a confirmed TB case count of 5 or more, and the presumptive to confirmed case count was less than 10. Similar alarm thresholds were set for individual states. The thresholds that drove the state algorithms were informed by the historical state-level TB notification and epidemiological data. The EWORS applied the incoming ward level data against these thresholds to determine wards and communities that met the thresholds and automatically generated alarms for affected communities. The algorithms analyzed data on a rolling 7-day basis, and alarm notification emails were sent to field officers to institute community outreach activities. Following the notification, the state and local teams would review the alarms in concert with the heat maps, then mobilize and conduct mass TB screening outreach activities at the affected alarm locations. We identified 121 hot spot areas—an average of 10 (range 6-25) hot spots per state. Field officers used the World Health Organization (WHO) 4-symptom screen (W4SS) to offer TB screening to consenting individuals during community outreach activities. Symptomatic outreach participants (presumptive TB patients) were further evaluated for TB using the GeneXpert instrument or chest X-rays (for patients that could not produce sputum). Confirmed TB cases were linked to treatment and to the national TB program.

To demonstrate the effectiveness of the EWORS analytic system in identifying missing TB cases, this study compared the TB case yield from the hot spot wards and areas with the yield obtained from data from health outreach activities in 213 non–hot spot wards and areas in the same 14 project states. The non–hot spot areas were selected purposely from all the LGAs where one or more community hot spot areas were identified. The methods of TB screening, evaluation, notification, and treatment for consenting patients in the non–hot spot areas were the same as described for the hot spot areas.

Deidentified project data relevant for this report from the hot spots and non–hot spots were retrieved from the Commcare-based mobile app and analyzed retrospectively using SPSS (IBM Corp). The data included the participants’ age, sex, TB case yield, and the number needed to screen (NNS) for each location area (hot spot and non–hot spot). Proportions were compared with the chi-square test, and a *P* value of less than .05 was considered significant. TB case yield was operationally defined as TB cases detected directly through the mass TB screening activities, calculated as a percentage of persons screened versus presumptive TB cases within the defined geographical area.

To assess the effectiveness of EWORS, this study used hot spot analyses to verify whether there were statistically significant spatial clusters of confirmed TB cases. Getis-Ord Gi* and optimized hot spot analyses were conducted to reveal the spatial heterogeneity of hot spots and non–hot spots [13]. Additionally, we examined spatial-stratified heterogeneity, which indicated the spatial differences between multiple layers or domains [14]. Ignoring the characteristics of spatial-stratified heterogeneity in the analysis could have led to model misspecification and estimation errors [15]. In this study, we used the Q statistic to measure the degree of stratified heterogeneity for hot spot analysis. The Q statistic is a value of the intensity of spatially stratified heterogeneity. It ranges between 0 and 1 and is calculated by comparing the stratified heterogeneities within the attribute and those between the strata. The higher the Q value, the stronger the stratification heterogeneity effect [14]. The Q statistic values from the Getis-Ord Gi* and optimized hot spot analyses were compared to each other to select the best hot spot model, which was then used to estimate the effectiveness of the EWORS. The Q statistic was calculated using the R package *geodetector*.

### Ethical Considerations

The study was determined to be a nonresearch program evaluation. It required no direct contact with human subjects (ie, there were no interviews and no sample collection). Also, only deidentified, pooled program data that formed part of the standard of care were used; thus, informed consent was not required.

## Results

**Figure 1:**
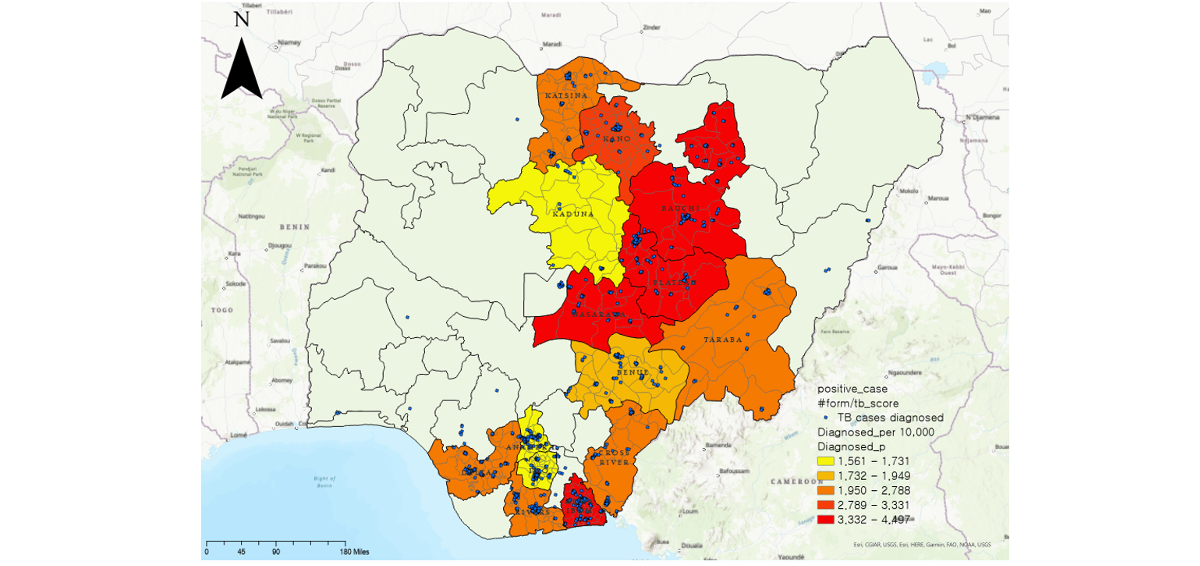
Tuberculosis cases diagnosed per 10,000 people. Blue dots indicate diagnosed patients. Colors closer to red indicate a higher number of tuberculosis cases diagnosed per 10,000 people. Created using ArcGIS® software by Esri and are used under license. Copyright © Esri.

To identify TB hot spots in the 14 study states, we first mapped them based on the number of diagnosed TB cases per 10,000 people. The result is shown in Figure 1. As shown on the map, Plateau, Nasarawa, and Akwa Ibom states had the highest numbers of confirmed cases. On the other hand, the states of Imo, Kaduna, and Anambra showed relatively low numbers.

Table 1 shows the estimated numbers of cases, TB tests, and treatments in the 14 states based on the numbers of diagnosed TB cases per 10,000 people. These results confirmed that to accurately identify hot spots and ultimately evaluate the effectiveness of the EWORS, it would be necessary to consider various factors, such as the number of presumptive TB cases and disease density, as well as the number of TB diagnoses in each state.

**Figure 2:**
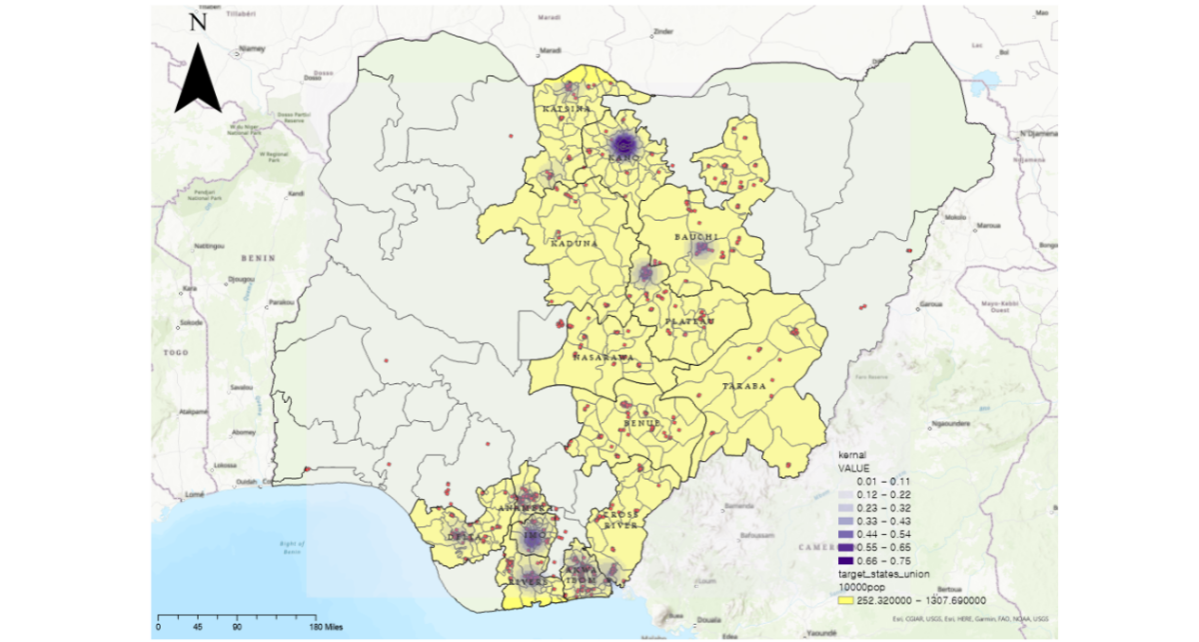
Kernel density based on number of diagnosed tuberculosis cases. Darker shades of purple indicate a higher density of occurrences. Created using ArcGIS® software by Esri and are used under license. Copyright © Esri.

TB is a representative infectious disease, so to find precise hot spots, we considered the number of occurrences and the density of diagnosed cases. Therefore, we performed a kernel density analysis based on the location where TB was diagnosed. This analysis differentiated between regions with a high number of diagnosed TB cases but low disease density and areas with a low number of diagnosed cases but high disease density. Using a threshold for kernel density of 46,242.3 meters, we applied planar kernel density estimation for mapping. In planar kernel density estimation, an area is characterized as a 2D, uniform Euclidean space, and its density is estimated at many regularly spaced positions [16,17]. Figure 2 shows the results of the kernel density analysis. The density of TB was relatively high in the northern and southern regions but with different characteristics. The northern region showed a very high density only in a specific area centered on Kano state. On the other hand, TB cases were widely distributed in the southern regions with a relatively low density. We defined TB hot spots by considering the number and density of hot spots using the optimal hot spot analysis technique with LGAs as units.

The Moran I test revealed a statistically significant level of spatial autocorrelation among confirmed tuberculosis cases (Moran I=0.773, *z* score=31.54), which confirms the validity of the hot spot analysis. Table 2 shows the results of the Getis-Ord Gi* and optimized hot spot analyses, indicating that the Getis-Ord Gi* method had a higher spatial stratified heterogeneity measured by the Q statistic than the optimized method. Therefore, in this study, the effectiveness of the EWORS was estimated using the Getis-Ord Gi* method.

**Table 1:** Ranking of the 14 study states for number of screened tuberculosis cases. All values represent cases per 10,000 people.

	State	Screened, n	Presumptive, n	Tested, n	Diagnosed, n	Treated, n
1	Plateau	957.29	50.16	38.71	4.25	4.08
2	Taraba	696.20	55.08	41.38	5.23	4.62
3	Bauchi	667.17	38.10	25.90	3.91	3.86
4	Nasarawa	598.21	44.80	34.50	3.47	3.31
5	Delta	585.78	34.05	28.40	2.63	2.34
6	Katsina	487.82	29.69	26.68	2.36	2.15
7	Cross River	460.68	32.18	25.52	2.25	2.06
8	Kano	442.55	33.01	27.61	3.01	2.90
9	Akwa Ibom	394.03	27.07	22.32	3.96	3.42
10	Anambra	360.45	38.01	30.04	1.61	1.52
11	Rivers	347.40	25.67	22.17	2.16	1.90
12	Kaduna	343.31	21.20	15.97	1.56	1.52
13	Imo	325.84	19.89	15.15	1.48	1.43
14	Benue	315.41	22.31	17.33	1.57	1.46

**Table 2:** Results for the Q statistic.

Hot spot model			Value		*P* value
**Optimized method**					
	*F*	404.88		<.001	
	Q statistic	0.603		<.001	
Getis-Ord Gi* method					
	*F*	251.61		<.001	
	Q statistic	0.709		<.001	

**Figure 3:**
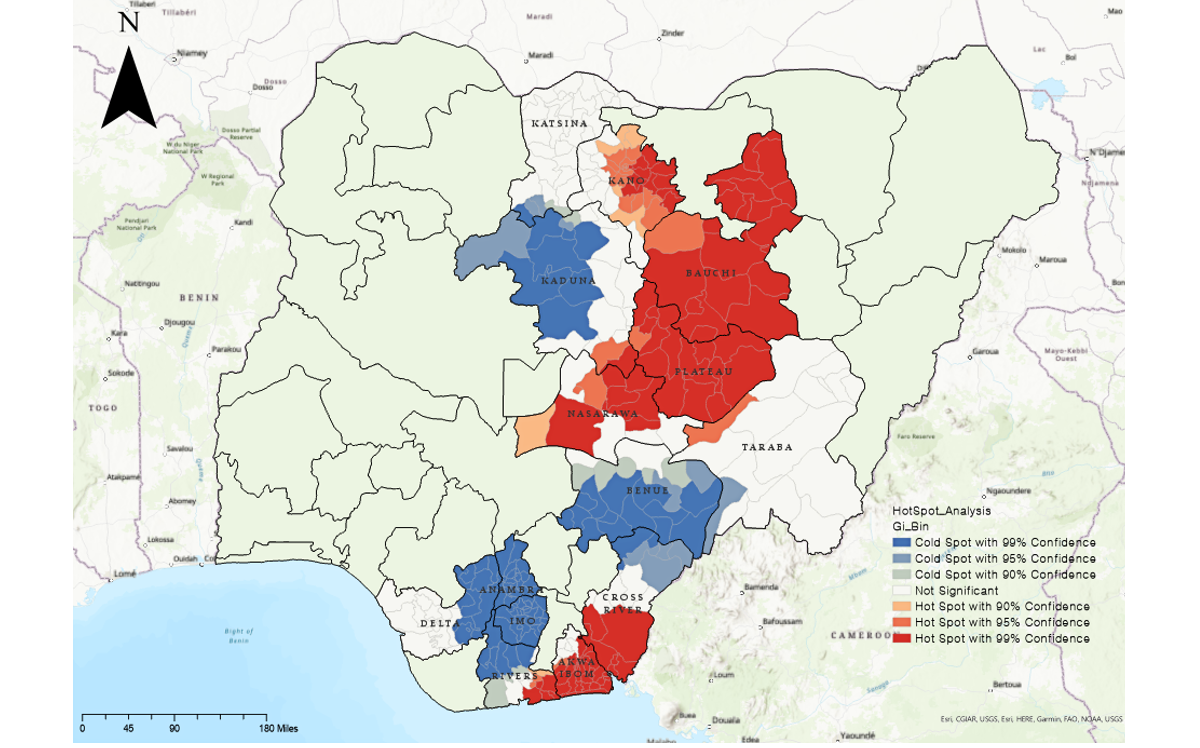
Hot spot analysis of diagnosed tuberculosis cases per 10,000 people. Red areas on the map indicate hot spots discovered through the early warning outbreak recognition system and blue areas indicate cold spots. Created using ArcGIS® software by Esri and are used under license. Copyright © Esri.

Figure 3 shows the hot spot identification results. The hot spot areas had a higher TB incidence with a higher density than the surrounding area. On the other hand, the cold spots were areas with a lower occurrence frequency and density than the surrounding area. The unmarked areas indicate that there might have been confirmed cases, but the hot spot clustering was not statistically significant.

With the EWORS, we were able to identify 121 hot spots in LGAs, 113 cold spots, and 100 other regions, but cold spots and other areas were combined as “non–hot spot” areas for analysis in this study. Therefore, we identified 121 hot spot regions and 213 non–hot spot regions to confirm the effectiveness of the EWORS. We first examined the characteristics of the TB outbreaks in hot spots and non–hot spots. The results are shown in Table 3. In the TB case-finding intervention, 1,962,042 persons (n=734,384, 37.4% male, n=1,227,658, 62.6% female) versus 2,025,286 persons (n=701,103, 34.6% male, n=1,324,183, 65.4% female) participated in the community TB screening outreaches in the hot spot and non–hot spot areas, respectively. The participants’ most and least common age groups for both areas were 25 to 34 years (hot spots: 447,911/1,962,042, 22.8%; non–hot spots: 506,913/2,025,286, 25%) and 65 years or older (hot spots: 117,212/1,962,042, 6%; non–hot spots: 133,360/2,025,286, 6.6%). Only 127,733 of 1,962,042 (6.5%) patients in the hot spot areas were presumptive TB cases, against 140,531 of 2,025,286 (6.9%) patients in the non–hot spot areas. Details of the distribution of participants in both hot spot and non–hot spot regions according to sex, age group, and number of presumptive TB cases identified and evaluated for TB are shown in Table 3.

As shown in Table 4, of the 3,987,328 patients screened, 222,270 (ie, 5.5% of all patients screened during the project) were further evaluated for TB in the hot spot and non–hot spot areas. The patients were predominantly female (n=2,551,841, 64%) and the modal age group for presumptive TB cases tested was 25 to 34 years (n=954,824, 23.9%). A total of 22,618 patients were confirmed TB cases, giving a TB yield of 10.2% (n=222,270) among presumptive TB cases evaluated and a yield of 0.6% (3,987,328) among all patients screened for TB. Among the TB cases, males predominated (n=13,607, 60.2%) while the main age group was 25 to 34 years (n=5,325, 23.5%). Out of all TB cases diagnosed, 21,071 (93.1%) were placed on treatment.

**Table 3:** Distribution of participants by sex, age group, and number of presumptive tuberculosis cases in hot spot and non–hot spot areas.

Characteristics			Hot spot area patients (n=1,962,042)		Non–hot spot area patients (n=2,025,286)
**Sex, n (%)**					
	Male	734,384 (37.4)		701,103 (34.6)	
	Female	1,227,658 (62.6)		1,324,183 (65.4)	
**Age group (years), n (%)**					
	0-4	211,792 (10.8)		258,942 (12.8)	
	5-14	169,125 (8.6)		130,761 (6.5)	
	15-24	329,470 (16.8)		282,060 (13.9)	
	25-34	447,911 (22.8)		506,913 (25)	
	35-44	326,720 (16.7)		350,993 (17.3)	
	45-54	214,122 (10.9)		214,544 (10.6)	
	55-64	145,690 (7.4)		147,713 (7.3)	
	≥65	117,212 (6)		133,360 (6.6)	
**Presumptive TB^a^ cases, n (%)**					
	Identified TB	127,733 (6.5)		140,531 (6.9)	
	Tested for TB	108,281 (5.5)		113,989 (5.6)	

^a^TB: tuberculosis.

**Table 4:** Patients screened for tuberculosis, presumptive tuberculosis cases evaluated, and diagnostic outcomes in the hot spot and non–hot spot areas.

Characteristic		Patients screened (N=3,987,328)	Presumptive TB^a^ cases evaluated (222,270/3,987,328, 5.5%)	All TB cases diagnosed (22,618/222,270, 10.1%)	All TB cases treated (21,071/22,618, 93.1%)
**Sex, n (%)**					
	Male	1,435,487 (36)	97,171 (43.7)	13,607 (60.2)	12,762 (60.6)
	Female	2,551,841 (64)	125,099 (56.3)	9011 (39.8)	8309 (39.4)
**Age groups (years), n (%)**					
	0-4	470,734 (11.8)	16,366 (7.4)	608 (2.7)	552 (2.6)
	5-14	299,886 (7.5)	18,309 (8.2)	1142 (5)	1062 (5)
	15-24	611,530 (15.3)	31,962 (14.4)	3472 (15.4)	3286 (15.6)
	25-34	954,824 (23.9)	47,202 (21.2)	5325 (23.5)	4945 (23.5)
	35-44	677,713 (17)	40,612 (18.3)	4884 (21.6)	4487 (21.3)
	45-54	428,666 (10.8)	27,281 (12.3)	3142 (13.9)	2941 (14)
	55-64	293,403 (7.4)	20,094 (9)	2102 (9.3)	1982 (9.4)
	≥65	250,572 (6.3)	20,444 (9.2)	1943 (8.6)	1816 (8.6)

^a^TB: tuberculosis.

Table 5 compares the TB diagnostic outcomes per 10,000 people between the hot spot and non–hot spot areas based on the EWORS. We used precision, number needed to screen (NNS), and number needed to test (NNT) to examine the effectiveness of TB diagnosis in the EWORS hot spot and non–hot spot areas. Precision is the ratio of true diagnosed cases to total presumptive cases; the higher the value, the higher the diagnostic accuracy. On the other hand, NNS and NNT refer to the ratios required for screening and testing for accurate diagnoses. Therefore, the lower the value, the lower the social cost. The prevalence of TB among all patients in the hot spot areas (3.76 per 10,000) was significantly higher than that in non–hot spot areas (2.14 per 10,000, *P*<.001; 95% CI 0.96-2.45). The precision was 10.5% for hot spots and 7.5% for the non–hot spots. The NNS for the hot spot areas was lower than the non–hot spot areas (146.22 versus 193.44). The NNT required fewer cases in hot spots, with an NNT in hot spots and non–hot spots of 9.51 and 13.43, respectively.

**Figure 4:**
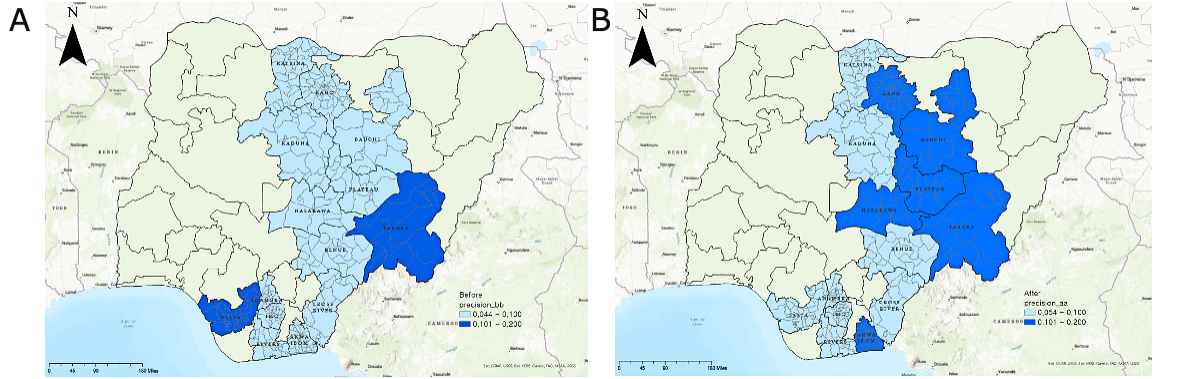
Comparison of diagnostic precision before and after application of an early warning outbreak recognition system. Light blue indicates precision less than 10%, and dark blue indicates precision higher than 10%. A: Before the system was applied (average precision: 7.7%); B: after the system was applied (average precision: 10.3%). Created using ArcGIS® software by Esri and are used under license. Copyright © Esri.

We thus confirmed that the EWORS could effectively identify TB hot spots and cold spots. According to the EWORS, the number of confirmed cases of TB per 10,000 people was higher in hot spot areas. Higher accuracy and a lower NNS and NNT were needed for hot spot areas with a higher risk of TB transmission. Based on these results, we compared the changes before and after application of the EWORS to measure its effectiveness, with particular attention to its effectiveness based on an analysis of the changes in precision and NNT before and after EWORS was applied in 14 major states. A 1-tailed, paired *t* test was used as an analysis method. Figure 4 shows the precision before and after EWORS application. According to the map results, before EWORS was applied, the average precision was 7.7% and was less than 10% in all regions. On the other hand, the precision after applying EWORS increased to an average of 10.3%. In other words, in the region where EWORS was applied, the precision to identify actual TB cases increased significantly.

**Table 5:** Comparison of tuberculosis diagnostic outcomes between the hot spot and non–hot spot areas.

	Hot spot area	Non–hot spot area	*P* value
Patients screened per local government area (per 10,000 people), n	549.80	413.97	N/A^a^
Presumptive cases evaluated for tuberculosis (per 10,000 people), n	35.79	28.75	N/A
All tuberculosis cases diagnosed (per 10,000 people), n	3.76	2.14 (95% CI 0.96-2.45)	<.001
Precision	0.105	0.075	N/A
Number needed to screen (per 10,000 people), n	146.22	193.44	N/A
Number needed to test (per 10,000 people), n	9.51	13.43	N/A

^a^N/A: not applicable.

Table 6 shows the paired *t* test results for changes in precision before and after the EWORS was applied. Comparing the overall scores shows that the precision before the EWORS was applied was 7.7%. However, after applying the EWORS, the precision was 10.3%. This was a statistically significant improvement of about 2.7%. We also compared the precision changes by dividing the hot spot and non–hot spot regions. Prior to the application of the EWORS, the precision of the hot spot areas was 6.4%. However, after applying the system, the accuracy of the actual TB diagnoses increased to 11.9%. Similarly, in non–hot spot regions, the precision increased to 9.2% after the EWORS was applied. In other words, it was possible to improve the precision of diagnosing cases of TB among presumptive cases with the EWORS.

**Figure 5:**
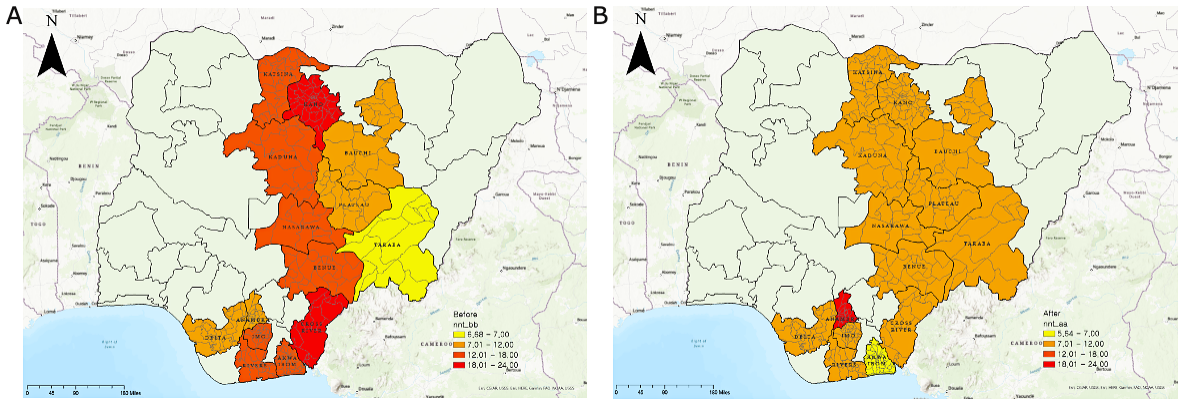
Comparison of numbers needed to test for tuberculosis diagnosis before and after the early warning outbreak recognition system was applied. Deeper shades of red indicate a higher number. A: Before the system was applied (average number needed to test: 14.047); B: after the system was applied (average number needed to test: 10.255). Created using ArcGIS® software by Esri and are used under license. Copyright © Esri.

Next, we compared the NNT for diagnosing TB among presumptive cases before and after the EWORS was applied. Figure 5 shows the NNT before and after the EWORS was applied. According to the figure, a high NNT was required in most states before the EWORS was applied. However, after the EWORS was applied, a very low NNT was needed in all but one state. In other words, NNT, representing the social cost of diagnosed cases, was found to be substantially reduced after the implementation of EWORS.

**Table 6:** The results of the paired *t* test for precision. Precision is defined as true positive cases / (correct presumptive cases + incorrect presumptive cases).

	Before the EWORS^a^	After the EWORS	Difference	*t_(668)_*	*P* value
Total precision (SE)	7.7% (0.12%)	10.3% (0.15%)	2.7% (0.19%)	13.764	<.001
Hot spot precision (SE)	6.4% (0.14%)	11.9% (0.26%)	5.5% (0.29%)	18.613	<.001
Non–hot spot precision (SE)	8.6% (0.15%)	9.2% (0.11%)	0.5% (0.19%)	2.747	.003

^a^EWORS: early warning outbreak recognition system.

Finally, we statistically compared the change in NNT before and after the EWORS was applied. The results are shown in Table 7. NNT before the EWORS was applied was 14.047 and decreased to 10.255 after it was applied, a statistically significant difference. Also, when the hot spot regions were classified, the NNT in the hot spot regions before the EWORS was applied was very high, at 16.788. However, after the system was applied, the NNT in the hot spot areas decreased to 8.829. Similarly, NNT was significantly reduced after using the EWORS in the non–hot spot regions. In other words, by applying the EWORS, NNT in the high-risk hot spot areas, as well as the entire area, was statistically significantly reduced.

**Table 7:** The results of paired *t* tests for the number needed to test to diagnose cases of tuberculosis among presumptive cases.

	Before the EWORS^a^	After the EWORS	Difference	*t_(668)_*	*P* value
Total number needed to test (SE)	14.047 (0.2229)	10.255 (0.1457)	3.792 (0.2663)	14.237	<.001
Hot spot number needed to test (SE)	16.788 (0.3756)	8.829 (0.1522)	7.958 (0.4053)	19.633	<.001
Non–hot spot number needed to test (SE)	12.006 (0.1498)	11.316 (0.1953)	0.690 (0.2462)	2.802	.004

^a^EWORS: early warning outbreak recognition system.

## Discussion

KNCV Nigeria proposed that an EWORS, which is primarily used to detect and control infectious-disease outbreaks, could predict TB hot spots at the ward and community levels by identifying TB patients’ residences in clusters. This project made innovative use of an EWORS for identifying TB hot spot areas in 14 states of Nigeria and found that the TB case yield in hot spot areas was significantly higher than in non–hot spot areas. Such large-scale, community-based ACF was necessary to counter the negative effect of the COVID-19 pandemic on TB notification and care at both the global and local levels [2,4]. Despite the predominance of female patients during outreach, there was a higher predominance of males among the TB cases, as expected because of the known epidemiology of TB in Nigeria [18-20]. The predominant age group also agreed with the reported pattern in the Nigerian population [7,18,21].

The WHO recommends systematic screening for TB among the general population in areas with an estimated TB prevalence of 0.5% or higher [22]; therefore, with an average TB case yield per LGA of 3.8% in the hot spot areas and 2.14% in the non–hot spot areas, the ACF interventions were justified in all areas of the project states. Moreover, this project demonstrated that because the EWORS tracked clusters of households with TB cases, it offered the added advantage of predicting communities with higher than usual TB prevalence (ie, hot spot areas), which will help prioritize community TB screening activities in underresourced settings like Nigeria. Despite the relatively lower efficiency of the W4SS TB screening method in the general population, it was suitable for this large-scale project because it is easy to implement, cheap, highly acceptable to patients, and accurate [22]. However, with a sensitivity of about 71%, a few false-negative TB cases were likely missed [20]. The same algorithm was used for both hot spot and non-spot areas, so the effect of this limitation on the study’s estimates would have been nondirectional. Nevertheless, with a reported sensitivity of 85% to 94%, mobile chest X-rays for community ACF, where affordable, would have reduced the missed cases and increased the TB yield for both areas [22].

The average precision and diagnostic accuracy for identifying a patient as a TB case following W4SS TB screening and further testing with GeneXpert instrument/chest X-ray in the EWORS-identified hot spot areas was about twice that of the non–hot spot areas in this project (Table 5). This finding suggests that the EWORS can be used to enhance the identification of missed TB cases in Nigerian communities. Most importantly, in underresourced settings such as Nigeria, it can be used to prioritize the siting of community health outreaches for TB to save cost. The EWORS was designed as an early warning system to detect infectious disease outbreaks and guide control practices, so its adaptation in this project as a warning system for areas or wards with a high cluster of TB cases, marking them for follow-up with community ACF, was innovative and novel. The possible effect of the W4SS TB screening regarding missed cases discussed above was a limitation. Also, the project did not evaluate patients without TB symptoms for TB preventive treatment eligibility.

The analysis showed that the EWORS effectively identified tuberculosis-risk hot spots in the 14 Nigerian states. In particular, it offered high precision, a low NNS, and a low NNT for hot spot areas. We also measured the effectiveness of applying the EWORS and found that the precision increased significantly after it was applied. In particular, the precision of hot spots was dramatically improved, and the number of presumptive cases required for a diagnosis of TB saw a statistically significant decrease after the EWORS was applied. Taken together, the EWORS not only effectively identified hot spots but also improved precision after it was applied. Prior to the introduction of the EWORS, the TB surveillance process to identify hot spots in Nigeria involved the manual collation of TB notification data by a designated local government TB and leprosy supervisor (LGTBLS) at the LGA level. Each LGTBLS collated data from each health facility within the LGA on case findings at the end of the quarter and submitted the data to the state TB program; thereafter, the data were reviewed to determine areas of high or low TB notification [23].

The EWORS applies a syndromic surveillance-based EWS and uses kernel density estimation to create a smooth, continuous surface for the density of observations. The application of Bayesian techniques in EWORS hot spot identification helps to reduce the rates of false-negative and false-positive hot spots in comparison to classical methods of hot spot identification, such as confidence intervals [24]. This is in line with other recent tools, such as Gettis-Ord Gi*, StatsCan, and Local Indicators of Spatial Association (LISA), which are used to improve the identification of hot spots through the “mining” of spatial patterns and applying hot-spot related factors, which can help distinguish between hot spots and normal areas [25]. However, while the Gettis-Ord Gi* and StatsCan add definition to maps by estimating the density distribution of events at the local level, which allows assessment of the spatial association of a particular observation or in a study area and identifies statistically significant hot spots and cold spots, the LISA technique measures the extent to which points that are close to a given point have similar values based on a measure of contiguity among these units within a specified radius, and thus is useful for identifying local spatial autocorrelation [26]. These methods serve as complementary techniques to address the limitations of mapping methods that use geographic boundary areas, such as uniform grid cells (or quadrants) or census blocks as the unit of spatial analysis (eg, thematic maps) to depict patterns of spatial clustering [24].

However, our approach has some limitations. First, we used planar kernel density estimation to show the density of tuberculosis outbreaks by region. Since the planar method assumes spatial homogeneity, it is impossible to confirm the movement of patients with confirmed tuberculosis over time [16]. Also, we used the optimal threshold for kernel density mapping, but there may be differences in the density map depending on the choice of reference point. In addition, we analyzed hot spots by LGA unit, but aggregated them at the state level when comparing efficiencies. This approach may find hot spots more accurately by considering internal deviations within each state, but the efficiency comparison is restricted to only the state level. Additionally, our hot spot analysis results cannot be used to determine causation [17].

We conclude that active TB case finding in EWORS-mapped hot spot areas yielded a higher number of TB cases than in the non–hot spot areas in 14 high-burden states of Nigeria. As the national TB program scales up active TB case finding in Nigeria, the EWORS should be used to identify and prioritize wards or communities for TB screening health outreach to optimize the use of available resources.

## Abbreviations

ACF: active case finding
CIDARS: China Infectious Disease Automated Alert and Response System
EWORS: early warning outbreak recognition system
EWS: early warning systems
LGA: local government area
LISA: Local Indicator of Spatial Autocorrelation
NNS: number needed to screen
NNT: number needed to test
TB: tuberculosis
W4SS: WHO 4-symptom screen
WHO: World Health Organization
